# Patient-proxy agreement on change in acute stroke patient-reported outcome measures: a prospective study

**DOI:** 10.1186/s41687-021-00329-7

**Published:** 2021-07-06

**Authors:** Brittany R. Lapin, Nicolas R. Thompson, Andrew Schuster, Irene L. Katzan

**Affiliations:** 1 Quantitative Health Sciences, Lerner Research Institute, Cleveland Clinic, Cleveland, OH USA; 2 Center for Outcomes Research & Evaluation, Neurological Institute, Cleveland Clinic, Cleveland, OH 44195 USA

**Keywords:** Patient-reported outcome measures, Proxy, PROMIS, Agreement, Reliability, Stroke

## Abstract

**Objectives:**

Research has indicated proxies overestimate symptoms on patients’ behalves, however it is unclear whether patients and proxies agree on meaningful change across domains over time. The objective of this study is to assess patient-proxy agreement over time, as well as agreement on identification of meaningful change, across 10 health domains in patients who underwent acute rehabilitation following stroke.

**Methods:**

Stroke patients were recruited from an ambulatory clinic or inpatient rehabilitation unit, and were included in the study if they were undergoing rehabilitation. At baseline and again after 30 days, patients and their proxies completed PROMIS Global Health and eight domain-specific PROMIS short forms. Reliability of patient-proxy assessments at baseline, follow-up, and the change in T-score was evaluated for each domain using intra-class correlation coefficients (ICC(2,1)). Agreement on meaningful improvement or worsening, defined as 5+ T-score points, was compared using percent exact agreement.

**Results:**

Forty-one patient-proxy dyads were included in the study. Proxies generally reported worse symptoms and functioning compared to patients at both baseline and follow-up, and reported less change than patients. ICCs for baseline and change were primarily poor to moderate (range: 0.06 (for depression change) to 0.67 (for physical function baseline)), and were better at follow-up (range: 0.42 (for anxiety) to 0.84 (for physical function)). Percent exact agreement between indicating meaningful improvement versus no improvement ranged from 58.5–75.6%. Only a small proportion indicated meaningful worsening.

**Conclusions:**

Patient-proxy agreement across 10 domains of health was better following completion of rehabilitation compared to baseline or change. Overall change was minimal but the majority of patient-proxy dyads agreed on meaningful change. Our study provides important insight for clinicians and researchers when interpreting change scores over time for questionnaires completed by both patients and proxies.

## Introduction

Multiple domains of health are impacted in patients with stroke including physical health, fatigue, pain interference, cognitive function, and overall global health [1]. Patient-reported outcome measures (PROMs) are increasingly utilized as endpoints for assessing these areas which are best evaluated through self-report. One challenge in the interpretation of PROMs is when caregivers, or proxies, respond instead of the patient, which can occur for as many as 30% of stroke patients [2, 3]. Research has indicated proxies overestimate symptoms on patients’ behalves, and this overestimation is greater for more subjective domains such as emotional or cognitive functioning [4, 5]. Patient-proxy disagreement has implications both for research studies and clinical care. In research studies, inclusion of unbalanced numbers of proxy respondents in different treatment groups may bias analyses of outcomes. At the patient-level, this disagreement could affect the clinical treatment of symptoms, which could differentially impact more subjective domains such as anxiety or depression.

Prior work by our group has demonstrated patient-proxy disagreement results in small effect sizes for group-level analyses, but large meaningful differences at the individual-level which affects the interpretability, and thus utilization, of PROMs during clinical care [6]. Furthermore, it is unclear whether patients and proxies agree on meaningful change across domains over time. Prior research evaluating stroke patient-proxy agreement on PROMs over time has been limited and results have been inconsistent, with one study finding low agreement between change scores [7] and another finding moderate agreement [8]. To our knowledge, no studies have investigated patient-proxy agreement on detecting meaningful change.

The objective of this study is to expand upon previous work and assess patient-proxy agreement over time, as well as agreement on identification of meaningful change, across 10 health domains in patients who underwent acute rehabilitation following stroke.

## Methods

Patients with ischemic stroke or intracerebral hemorrhage were recruited from an ambulatory clinic, an inpatient rehabilitation unit, and an outpatient rehabilitation unit. Patients were included in the study if they were currently undergoing or about to undergo rehabilitation, cognitively and physically able to complete questionnaires, and had a proxy available with them to answer questionnaires. Informed consent was obtained for participating patients and their proxy prior to clinic visit or during their rehabilitation admission. The full study protocol has been previously published [6]. Briefly, each proxy participant was instructed to answer the questions in the way they believed the patient would answer, according to the “patient-proxy” perspective [9]. For patients who were unable to complete the questionnaires at the time of the visit, surveys were collected by emails sent via REDCap electronic data capture tools [10]. Following completion of an initial set of surveys, patients and their proxies each received a $25 stipend for participation. Patients and proxies received an additional $15 stipend after completing a second set of the same surveys 30 days following completion of the initial surveys. Patients attended rehabilitation during this time, and it is anticipated that patients improved in these measured domains during this window.

As part of the questionnaire set at both time points, patients and proxies completed 9 PROMs: PROMIS Global Health (resulting in global mental and global physical health summary scores) and PROMIS 8-item short forms for physical function, satisfaction with participation in social roles and activities, anxiety, fatigue, pain interference, sleep disturbance, Neuro-QoL cognitive function, and the Patient Health Questionnaire 9 depression screen which was calibrated to the PROMIS Depression metric [11]. PROMIS measures are transformed to a T-score metric with a mean of 50 and standard deviation (SD) of 10, which is representative of the mean and SD of the general United Status population [12].

### Statistical analyses

Descriptive statistics were utilized to present patient and proxy characteristics, as well as responses to PROMs at baseline, follow-up, and change in PROM. Differences between patient versus proxy-reported PROM were compared using t-test. Significant change in PROM reported by patients and proxies was evaluated using paired t-test. Reliability of patient-proxy assessments at baseline, follow-up, and the change in T-score was assessed for each domain using intra-class correlation coefficients (ICC(2,1)) with 95% confidence intervals based on two-way random effects models for single rater agreement [13].

To identify agreement on meaningful improvement or worsening, a minimal important difference (MID) was calculated as half a SD, or 5 points [14, 15]. Agreement between patients and proxies reporting MIDs were compared using percent exact agreement and unweighted kappa with 95% CI. Analyses were conducted using R version 4.0.0. Statistical significance was established throughout at *p* < 0.05.

## Results

Forty-one patient-proxy dyads were included in the study with PROMs completed by both patients and their proxies at two time points (average ± sd 35.0 ± 13.9 days apart). The majority of patients were male (58.5%), white (85.4%), and married (85.4%), with average age 60.8 (±13.3) years (Table 1). Proxies were predominately female (78.0%) and spouses of the patient (73.2%).

**Table 1:** Baseline characteristics of patients and proxies

	Patients ***N*** = 41	Proxies *N* = 41
**Age**, mean (SD)	60.8 (13.3)	54.9 (13.4)
**Female**	17 (41.5%)	32 (78.0%)
**Race**		
White	35 (85.4%)	37 (90.2%)
Black	3 (7.3%)	4 (9.8%)
Other/missing	3 (7.3%)	0 (0.0%)
**Marital Status**		
Married/Partnered	35 (85.4%)	33 (80.5%)
Single	1 (2.4%)	4 (9.8%)
Divorced	2 (4.9%)	3 (7.3%)
Widowed	1 (2.4%)	1 (2.4%)
Missing	2 (4.9%)	0 (0.0%)
**Education**		
High School Graduate/GED	11 (26.8%)	9 (22.0%)
Some College	13 (31.0%)	14 (33.3%)
College	11 (26.2%)	12 (28.6%)
Graduate School	6 (14.3%)	7 (16.7%)
Missing	1 (2.4%)	0 (0.0%)
**Stroke Type**		
Ischemic	32 (78.0%)	
Intracerebral Hemorrhage	9 (22.0%)	
**mRS,** median (IQR)	2 (1, 3)	
**NIHSS Score**, median (IQR)	1 (0, 2.5)	
**Days Since Stroke,** median (IQR)	28 (17, 53)	
**Rehabilitation Location**		
Outpatient Therapy/Rehab	33 (80.5%)	
Inpatient Rehab	8 (19.5%)	
**Relationship to Patient**		
Spouse		30 (73.2%)
Parent		1 (2.4%)
Child		8 (19.5%)
Other Relative		2 (4.9%)
**Frequency of Contact w/ Patient**		
Live with		35 (85.4%)
Once per day		5 (12.2%)
Once per week		1 (2.4%)
Once per month or less		0 (0.0%)
**How Well Know Patient**		
Extremely		37 (90.2%)
Very		3 (7.3%)
Somewhat		0 (0.0%)
Slightly		1 (2.4%)
Not at all		0 (0.0%)

*SD* standard deviation, *IQR* interquartile range; Continuous variables that deviate from normality are presented with median and *IQR mRS* modified Rankin scale, *NIHSS* National Institutes of Health Stroke Scale

Proxies reported worse symptoms and functioning compared to patients on the domains of cognitive function, anxiety, depression, and fatigue at baseline, and on all domains but pain interference and sleep disturbance at follow-up (Table 2). These findings were statistically significant at baseline for the domain of cognitive function and at follow-up for the domains of cognitive function, global mental health, anxiety, and fatigue. Proxies typically reported less change than patients, with statistically significant proxy-patient differences on the domains of global mental health, social role satisfaction, and fatigue. Patients reported improvement on all domains, and significant improvement on 5 domains, compared to proxies who reported minimal change on domains and significant worsening on global mental health (− 2.6 T-score points).

**Table 2:** Means (standard deviations) of scales for patients and their proxies at baseline, follow-up, and change in score (follow-up – baseline), and intra-class correlation coefficients

Domain	Visit	Self-Reported Mean (SD)	Proxy-Reported Mean (SD)	Difference (Proxy – Self-Report) Mean (SD)	ICC (95% CI)
**Cognitive Function,** ***n*** **= 40**	Baseline	46.9 (9.9)	43.8 (9.5)	− 2.6 (8.1)*	0.63 (0.44, 0.76)
	Follow-up	47.4 (9.2)	43.8 (10.1)	−3.5 (7.4)*	0.67 (0.46, 0.80)
	Change	0.8 (8.7)	0.0 (8.1)	−0.8 (10.1)	0.29 (0.04, 0.51)
**Physical Function,** ***n*** **= 41**	Baseline	36.0 (9.8)	36.6 (10.3)	0.6 (8.3)	0.67 (0.50, 0.79)
	Follow-up	38.6 (11.7)	37.4 (10.7)	−1.2 (6.4)	0.84 (0.74, 0.90)
	Change	2.7 (5.2)ƚ	0.8 (10.1)	−1.8 (8.6)	0.42 (0.18, 0.61)
**Global Mental Health,** ***n*** **= 41**	Baseline	46.2 (8.8)	46.8 (9.0)	0.6 (9.4)	0.45 (0.22, 0.64)
	Follow-up	48.1 (9.0)	44.2 (10.1)	−4.0 (8.0)*	0.60 (0.37, 0.75)
	Change	1.9 (6.6)	−2.6 (6.6)ƚ	−4.6 (8.7)*	0.11 (− 0.10, 0.32)
**Global Physical Health,** ***n*** **= 41**	Baseline	41.4 (7.6)	41.6 (7.7)	0.2 (7.8)	0.48 (0.26, 0.66)
	Follow-up	44.5 (8.8)	42.9 (8.6)	−1.7 (5.4)	0.80 (0.68, 0.88)
	Change	3.1 (7.3)ƚ	1.3 (8.0)	−1.8 (7.4)	0.53 (0.32, 0.69)
**Social Role Satisfaction,** ***n*** **= 41**	Baseline	40.8 (9.2)	42.5 (9.0)	1.7 (9.7)	0.42 (0.19, 0.61)
	Follow-up	44.9 (10.0)	43.3 (11.5)	−1.7 (10.7)	0.51 (0.29, 0.68)
	Change	4.1 (5.9)ƚ	0.7 (10.2)	−3.4 (10.3)*	0.21 (− 0.03, 0.44)
**Anxiety**ǂ**,** ***n*** **= 41**	Baseline	51.4 (7.4)	54.5 (9.2)	3.1 (10.0)	0.27 (0.03, 0.48)
	Follow-up	47.8 (8.9)	52.1 (8.8)	4.3 (9.1)*	0.42 (0.19, 0.62)
	Change	−3.6 (9.2)ƚ	−2.4 (10.6)	1.2 (10.6)	0.43 (0.20, 0.62)
**Depression**ǂ**,** ***n*** **= 40**	Baseline	52.8 (8.1)	54.7 (9.2)	1.9 (11.7)	0.09 (− 0.17, 0.34)
	Follow-up	51.4 (8.8)	53.0 (9.0)	1.8 (8.1)	0.58 (0.38, 0.73)
	Change	−1.1 (8.1)	−1.7 (10.1)	− 0.5 (12.7)	0.06 (− 0.20, 0.31)
**Fatigue**ǂ**,** ***n*** **= 41**	Baseline	57.6 (7.6)	57.9 (7.5)	0.3 (9.3)	0.26 (0.00, 0.48)
	Follow-up	53.0 (9.3)	56.9 (9.7)	3.8 (9.1)*	0.51 (0.28, 0.68)
	Change	−4.6 (7.1)ƚ	−1.0 (9.8)	3.6 (10.7)*	0.20 (− 0.04, 0.43)
**Pain Interference**ǂ**,** ***n*** **= 41**	Baseline	52.3 (10.0)	51.5 (9.6)	−0.7 (8.8)	0.60 (0.41, 0.74)
	Follow-up	50.9 (10.5)	50.6 (9.6)	−0.3 (7.5)	0.73 (0.58, 0.83)
	Change	−1.4 (8.4)	−0.9 (9.6)	0.5 (9.3)	0.47 (0.25, 0.65)
**Sleep Disturbance**ǂ**,** ***n*** **= 40**	Baseline	51.8 (8.4)	51.3 (10.5)	−0.5 (8.1)	0.65 (0.47, 0.78)
	Follow-up	50.2 (9.7)	49.4 (10.3)	−0.3 (9.7)	0.54 (0.33, 0.70)
	Change	−2.0 (10.7)	−1.9 (9.1)	0.1 (10.0)	0.50 (0.28, 0.67)

*p < 0.05 for between group (patient vs proxy) difference based on t-test at baseline, follow-up, or change in scores. ƚ*p* < 0.05 for within group (patient or proxy) change between baseline and follow-up based on paired t-test. ǂHigher scores indicate worse symptoms/functioning; ICC = intra-class correlation coefficient with 95% confidence interval (CI) based on a two-way random effects model for single rater consistency. An ICC of < 0.40, 0.41–0.60, 0.61–0.80, and > 0.80 indicates poor, moderate, substantial, and high reliability, respectively

At baseline, ICCs were poor to substantial, ranging from 0.09 for depression to 0.67 for physical function (Table 2). Compared to baseline, reliability was better at follow-up for all domains except sleep disturbance, and ranged from 0.42 for anxiety to 0.84 for physical function. Compared to baseline and follow-up, agreement with determining change had the lowest ICCs for the majority of domains, and was generally poor, ranging from 0.06 for depression to 0.53 for global physical health.

**Fig. 1:**
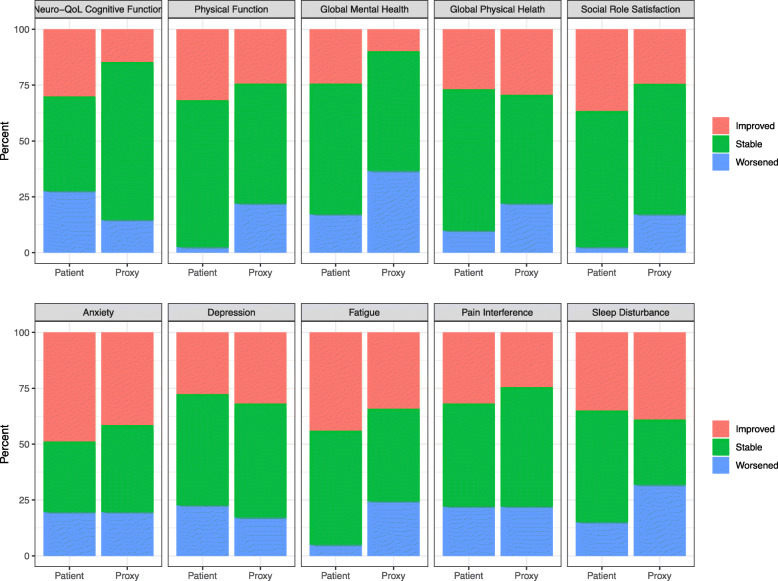
Comparison of change by patient/proxy report between all patient-proxy dyads, *n* = 41. Improved = improvement of 5+ T-score points; Worsened = worsening of 5+ T-score points; Stable = change within 5 T-score points

Based on MIDs, patients indicated more meaningful improvement than proxies across the majority of domains (Fig. 1). The number of patient-proxy dyads that both indicated meaningful improvement ranged from 2 (4.9%) for global mental health to 11 (26.8%) for anxiety (Table 3). Percent exact agreement between indicating meaningful improvement versus no improvement was fairly high, from 58.5% for social role satisfaction to 75.6% for global mental health. Based on the kappa statistic, agreement between dyads on meaningful improvement was generally slight, with the lowest agreement on the domains of social role satisfaction and depression (kappa statistic = 0.04 and 0.05, respectively) (Table 3). The highest agreement was on the domains of sleep disturbance, pain interference, and physical function (kappa = 0.47, 0.34, and 0.34, respectively).

**Table 3:** Number of patients and proxies reporting meaningful improvement or worsening from baseline to follow-up, percent exact agreement, and kappa statistic

Domain	Improvement			Worsening		
	Dyads indicating meaningful improvement, n (%)	% Exact Agreement	Kappa (95% CI)	Dyads indicating meaningful worsening, n (%)	% Exact Agreement	Kappa (95% CI)
Cognitive Function	3 (7.5%)	70.0%	0.17 (− 0.14, 0.48)	3 (7.5%)	72.5%	0.20 (− 0.13, 0.52)
Physical Function	6 (14.6%)	73.2%	0.34 (0.03, 0.65)	0 (0.0%)	75.6%	−0.05 (− 0.13, 0.04)
Global Mental Health	2 (4.9%)	75.6%	0.17 (−0.15, 0.49)	4 (9.8%)	65.9%	0.17 (−0.11, 0.46)
Global Physical Health	5 (12.2%)	68.3%	0.22 (−0.11, 0.54)	2 (4.9%)	78.0%	0.20 (−0.14, 0.54)
Social Role Satisfaction	4 (9.8%)	58.5%	0.04 (−0.26, 0.34)	0 (0.0%)	80.5%	−0.05 (− 0.12, 0.03)
Anxiety	11 (26.8%)	63.4%	0.27 (−0.03, 0.56)	4 (9.8%)	80.5%	0.38 (0.03, 0.73)
Depression	4 (10.0%)	60.0%	0.05 (−0.26, 0.36)	1 (2.5%)	65.0%	−0.09 (− 0.36, 0.18)
Fatigue	8 (19.5%)	61.0%	0.19 (−0.11, 0.49)	0 (0.0%)	70.7%	−0.09 (− 0.20, 0.02)
Pain Interference	6 (14.6%)	73.2%	0.34 (0.03, 0.65)	3 (7.3%)	70.7%	0.15 (−0.19, 0.48)
Sleep Disturbance	10 (25.0%)	75.0%	0.47 (0.19, 0.75)	2 (5.0%)	62.5%	0.01 (−0.27, 0.28)

Meaningful improvement or worsening = both patient and proxy indicated change from baseline to follow-up of at least 5 T-score points. Kappa interpretation: ≤0 = poor, .01–.20 = slight, .21–.40 = fair, .41–.60 = moderate, .61–.80 = substantial, and .81–1 = almost perfect

Overall, only a small proportion of patients and proxies indicated meaningful worsening on PROMs (Fig. 1). Less than 10 % of dyads designated meaningful worsening among the different domains (ranging from 0% for physical function, social role satisfaction, and fatigue to 9.8% for global mental health and anxiety) (Table 3). Percent exact agreement between patient and proxy scores on meaningful worsening ranged from 62.5–80.5%, although proxy agreement based on the kappa statistic was poor to slight for all domains except anxiety, which demonstrated moderate agreement on worsening (kappa = 0.38).

## Discussion

Our study assessed patient-proxy agreement, both over time and with identifying meaningful change, for 10 PROM domains in 41 patients who underwent rehabilitation following stroke. Patient-proxy agreement was better at follow-up compared to baseline or change, with higher agreement on more objective domains (ICC = 0.84 for physical function) and lower agreement on more subjective domains (ICC = 0.42 for anxiety). This is similar to a study of 164 stroke patients and their proxies where greater agreement was found on PROMs 6 months post-stroke compared to time of stroke [8]. Agreement was higher for more objective domains of ambulation/dexterity (ICCs = 0.75–0.87) and lower on more subjective domains such as hearing and cognition (ICCs = 0.20–0.31). In a study of 65 patient-proxy dyads, however, higher agreement was found at the time of stroke (ICCs> 0.69 for SF-12 physical and mental component scores) as compared to 6 months later or change in scores [7]. Generally, results from cross-sectional studies have been mixed when assessing patient-proxy agreement as time from stroke increases. Prior studies by our group have not shown an association between time from stroke and patient-proxy agreement [6, 16].

Overall, patients indicated significantly more improvement over time than proxies. Patient-proxy agreement on PROM change scores, as well as kappa statistics for assessing improvement, was better for more objective domains (physical function, global physical health, pain interference) and worse for more subjective domains (social role satisfaction, fatigue, depression, global mental health). When evaluating patient-proxy agreement on detecting worsening, there were minimal differences by domain, potentially owing to the low level of worsening overall. Similarly, the literature has indicated a lack of clinical change across health-related domains following stroke. Studies have shown that common post-stroke symptoms, such as fatigue, pain, anxiety, and depression, remain issues 6 months after stroke [17–19]. Minimal functional recovery has been demonstrated following mild stroke [20], and studies have indicated worse Neuro-QoL cognitive function scores at 3 months [21]. In our study, proxies indicated worse functioning and symptoms at follow-up and less change than patients. It has been posited that observers tend to place more weight on negative information than positive when providing impressions of others [22]. Our study is novel in that it evaluated patient-proxy agreement on reporting meaningful change. A prior study by our group found high levels of meaningful patient-proxy disagreement in a cross-sectional analysis, with 40–57% of dyads differing by 5+ T-score points across domains [6]. Our current study indicates patient-proxy agreement on meaningful change over time may be more reliable, as the majority of dyads agreed on meaningful improvement (59–76%) and worsening (63–81%) across domains. This has practical implications for the interpretation of assessing change in PROMs based on proxy-reports. Given the variability in patient-proxy disagreement at the individual-level in our prior study [6], and the current finding that proxies report worse scores and indicate less change than patients, it is unclear how reliable the clinical interpretation of a change in PROMs would be if patients answered at one time point and proxies at another. At a minimum, PROMs should include a question identifying whether they were completed by a patient or proxy, and clinicians should take this information into account when interpreting PROMs for use in clinical care.

There are limitations to our study, the most apparent being the small study sample. The full range of scores may not be observed in studies with small sample sizes, and patient-proxy agreement and correlations may be inflated by a few large standard errors [23]. Second, kappa statistics offer an added benefit of accounting for chance agreement [24], however they are limited when the marginal probability of one group is much smaller than the other [25]. Since the number of dyads indicating meaningful change was low in this study compared to dyads indicating no change, percent exact agreement may be more accurate than kappa statistics for assessing patient-proxy agreement. Third, there was variability in the amount of time that passed between the two assessments (range 17–93 days), further limiting the interpretation of the results. Fourth, our study sample was largely male, of White race, and married, which could limit generalizability of results. Lastly, our study did not include a clinical assessment for cognitive impairment and is limited to patients who were able to self-report their health status. Larger longitudinal studies over longer time periods that include clinical indicators are necessary to determine if proxies, and patients, can accurately assess meaningful change over time.

In conclusion, our study found patient-proxy agreement was better at follow-up in a study of 41 patient-proxy pairs who completed PROMs across 10 domains of health at baseline and again following completion of rehabilitation. When evaluating change, patient-proxy agreement on detecting improvement was better for more objective domains than more subjective domains. Although change was minimal, the majority of patient-proxy dyads agreed on meaningful improvement and worsening. Our study provides important insight for clinicians and researchers when interpreting change scores over time for PROMs completed by both patients and proxies.

## Data Availability

The dataset used during the current study is available from the corresponding author on reasonable request.

## Abbreviations

ICC: intra-class correlation coefficient
MID: minimal important difference
PROMs: Patient-reported outcome measures
SD: standard deviation
